# Primary omental torsion diagnosed and treated laparoscopically: a case report

**DOI:** 10.1093/jscr/rjab237

**Published:** 2021-06-04

**Authors:** Kentaro Imanishi, Norichika Iga, Daisuke Mizuno, Hideyuki Nishi, Shinichiro Miyoshi

**Affiliations:** Department of Surgery, Okayama Rosai Hospital, 1-10-25 Chikkomidorimachi, Minamiku, Okayama 702-8055, Japan; Department of Surgery, Okayama Rosai Hospital, 1-10-25 Chikkomidorimachi, Minamiku, Okayama 702-8055, Japan; Department of Surgery, Okayama Rosai Hospital, 1-10-25 Chikkomidorimachi, Minamiku, Okayama 702-8055, Japan; Department of Surgery, Okayama Rosai Hospital, 1-10-25 Chikkomidorimachi, Minamiku, Okayama 702-8055, Japan; Department of Surgery, Okayama Rosai Hospital, 1-10-25 Chikkomidorimachi, Minamiku, Okayama 702-8055, Japan

## Abstract

Omental torsion, a rare cause of acute abdomen in children and adults, is difficult to correctly diagnose before surgery because it mimics the common causes of acute surgical abdomen. We present a case of greater omental torsion that was diagnosed by laparoscopy. A 37-year-old man presented with right lower abdominal pain and was suspected to have appendicitis. Blood tests revealed elevated C-reactive protein and white blood cell count, whereas computed tomography revealed a nodular mass and high-density lesions in the fat tissue. As the patient’s abdominal symptoms were severe and a clear diagnosis could not be made, we performed exploratory laparoscopy. Laparoscopy revealed omental torsion, and an omentectomy was performed. The patient’s pain had significantly reduced post-surgery, and post-operative recovery was uneventful. Thus, laparoscopic examination is useful for accurately diagnosing omental torsion and is less invasive than surgery.

## INTRODUCTION

Torsion of the greater omentum is a rare acute abdominal condition that is difficult to distinguish from other acute abdominal pathologies. Additionally, omental torsion is difficult to accurately diagnose using blood tests and abdominal images, and abdominal pain often intensifies when the greater omentum is severely tortuous and obstructed. Exploratory laparoscopy is an optimal intervention, because it is less invasive than surgery and can be used to explore the cause of severe or worsening abdominal symptoms.

Herein, we report a case of greater omental torsion diagnosed by laparoscopy in an acute abdomen and treated appropriately.

## CASE REPORT

A 37-year-old man with no history of surgery presented to our hospital with suspected appendicitis. The patient had right lower abdominal pain 2 days before admission, and the pain was so severe that the patient was unable to maintain a neutral spine position.

**Figure 1:**
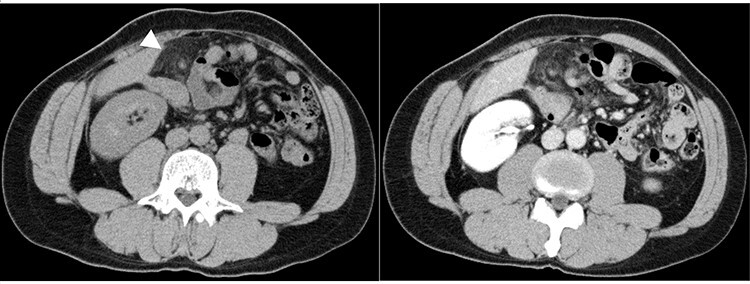
Abdominal CT images: nodular mass and high-density lesions in the fat tissue near the hepatic flexure of the ascending colon are evident (white arrow).

The patient’s body mass index was 23.5. On physical examination, the patient had a pulse of 73 beats/min, blood pressure of 133/82 mm Hg and temperature of 37.4°C.Rebound tenderness and abdominal guarding were recognized in the upper quadrant. The patient had no associated nausea or vomiting. Blood tests showed elevated C-reactive protein (CRP) and white blood cell (WBC) count (6.1 mg/dl and 9300/mm^3^, respectively). Plain abdominal computed tomography (CT) showed a nodular mass and high-density lesions in the fat tissue near the hepatic flexure of the ascending colon (Fig. 1). In contrast-enhanced CT, the nodular mass and torsion were unclear. Based on the CT scans and the patient’s severe clinical symptoms, acute appendicitis was considered doubtful; rather, we suspected acute abdominal conditions such as internal hernia, omental torsion, epiploic appendagitis, diverticulitis or omental infarction. Given the severity of the patient’s symptoms, observable deterioration and lack of a clear diagnosis, we performed exploratory laparoscopy.

**Figure 2:**
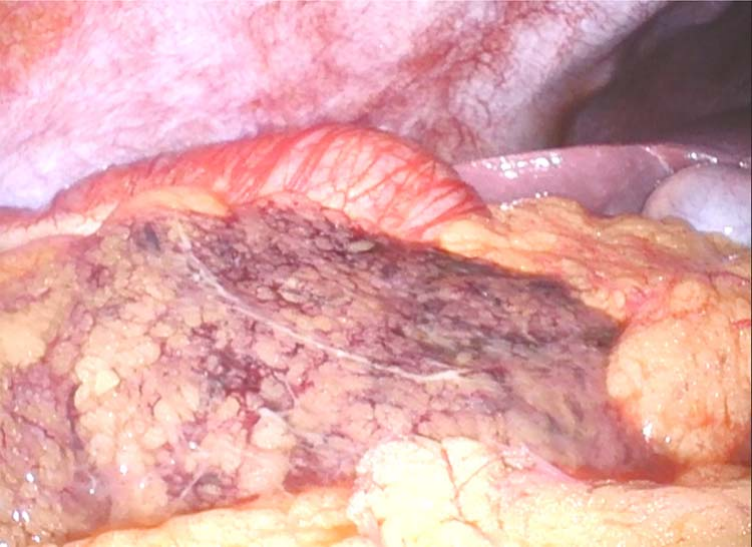
Ischaemic change is noted in the part of greater omentum that is adherent to the ascending colon near the hepatic flexure.

**Figure 3:**
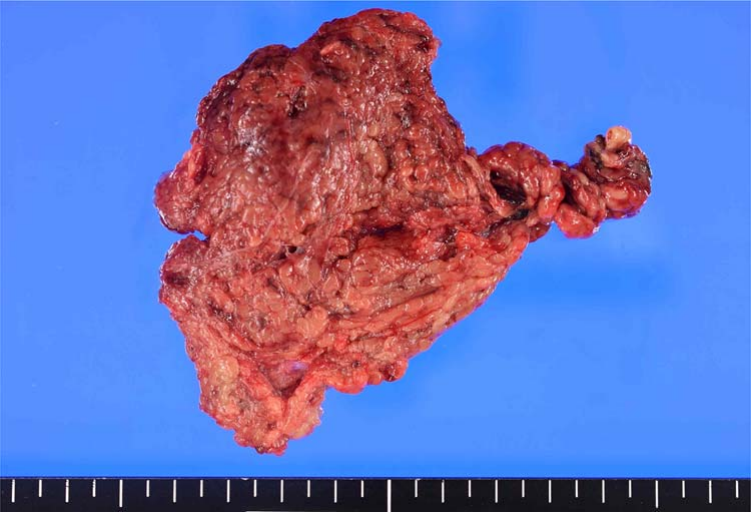
Macroscopic findings: the 10 cm × 7 cm-sized specimen is twisted and dark red in colour.

A 12-mm port was used to reach the abdominal cavity from the umbilicus, and two 5-mm trocars were placed in the right and left lower quadrants. Laparoscopy showed no ascites or peritonitis. The ischaemic change was recognized in the part of the greater omentum that adhered to the ascending colon near the hepatic flexure (Fig. 2). The adhesion around the necrotic lesion was resected, and the greater omentum was rotated several times around the vessel (Supplementary Video 1). The greater omentum in the ischaemic area was resected, and the specimen was retrieved through a small abdominal incision above the 12-mm trocar. No abnormalities were found in the appendix, gallbladder, small intestine or colon. The total operating time was 48 min. Macroscopically, the specimen was 10 cm × 7 cm in size and twisted (Fig. 3). Histology showed congestive and haemorrhagic lesions in the resected omentum. This confirmed the diagnosis of omental infarction due to omental torsion. The patient’s pain had significantly reduced post-surgery, and the patient was discharged from the hospital 5 days thereafter.

## DISCUSSION

Omental torsion is a rare cause of acute abdomen, accounting for 1.1% of acute abdomen cases underwent laparoscopy [1]. Omental torsion is caused by the twisting of the greater omentum around a pivotal point, which leads to impaired blood flow. Omental torsion is classified as either primary or secondary; primary omental torsion has no underlying pathologies, whereas secondary omental torsion is associated with adhesion, tumours, hernia or other pathologies [2]. Approximately 80% of omental torsion cases present with right lower abdominal pain [3]. The symptoms of omental torsion are similar to those of acute appendicitis and mimic acute pathologies such as bowel perforation, acute cholecystitis and Meckel’s diverticulitis. In women of reproductive age, differential diagnosis is needed to rule out ectopic pregnancy, salpingitis and ovarian cysts [4]. Patients with omental torsion often present with elevated CRP and WBCs, and some patients develop a fever. CT abdominal imaging is the gold standard for diagnosing omental torsion: The characteristic finding is a whirling pattern in the mesentery or fluid accumulation in the abdominal cavity [5]. The whirling pattern is useful for excluding differential diagnoses, such as appendicitis or cholecystitis. Another characteristic finding is hyperattenuated streaks of fat beneath the parietal peritoneum in the affected segment. However, only 0.6–4.8% of omental torsion cases have a preoperative or non-operative diagnosis; rather, most cases, including ours, are diagnosed intraoperatively [6].

The treatment of primary omental torsion is controversial. Conservative treatment has been recommended by many publications. However, the failure rate of conservative treatment is reportedly 15.9% and complications include unresolved pain, abscess formation and adhesions. Additionally, patients in whom conservative treatment failed and laparoscopic surgery was performed were more likely to require open surgery (27.2%), which was not observed in the surgically treated group [7]. Laparoscopy is a useful surgical strategy [8, 9] because precise diagnoses can be made by directly examining the abdominal cavity from a small incision. Additionally, laparoscopy is minimally invasive and, if necessary, allows quick transition to surgery. Since Chung *et al.* [10] reported the first laparoscopic management case, many surgeons advocate laparoscopy as the primary surgical approach for suspected omental torsion, especially when it is difficult to make a diagnosis before surgery. Moreover, initial management of patients with omental torsion results in a shorter hospital stay than conservative management (2 and 4 days, respectively) [11, 12]. In our case, the laparoscopic approach was selected, and the patient was diagnosed early. The patient was discharged shortly after the operation and made an uncomplicated recovery.

## CONCLUSION

Omental torsion often mimics other acute abdominal conditions; thus, it is difficult to diagnose. Laparoscopic examination is useful when diagnosis is doubtful or in cases with severe symptoms. Accordingly, we successfully treated a patient with omental torsion laparoscopically.

## Supplementary Material

Video_rjab237
